# Successful Treatment of Concomitant Pleural Mucosa-Associated Lymphoid Tissue Lymphoma and Monoclonal Gammopathy of Undetermined Significance with Lenalidomide, Rituximab, and Dexamethasone

**DOI:** 10.1155/2022/2027027

**Published:** 2022-05-30

**Authors:** Yoshiki Uemura, Risa Maeda, Hiroyoshi Saegusa

**Affiliations:** ^1^Department of Hematology, Chikamori Hospital, 1-1-16, Okawasuzi, Kochi-shi, Kochi-ken 780-8522, Japan; ^2^Department of Nephrology, Chikamori Hospital, 1-1-16, Okawasuzi, Kochi-shi, Kochi-ken 780-8522, Japan; ^3^Department of General Medicine, Chikamori Hospital, 1-1-16, Okawasuzi, Kochi-shi, Kochi-ken 780-8522, Japan

## Abstract

Concomitant plasma cell and B cell neoplasms in a single patient have been infrequently reported. It is known that the prognosis of these patients is worse than that of patients with single-disease onset. Generally, the chemotherapy specific for each disease is provided sequentially. It has been suggested that the specific chemotherapy for lymphoma could lead to the occurrence of refractory multiple myeloma (MM). We present a case with the concomitant occurrence of mucosa-associated lymphoid tissue (MALT) lymphoma and monoclonal gammopathy of undetermined significance (MGUS). MGUS does not usually require aggressive treatment. However, the potential adverse effects of MGUS on the treatment course of the B cell lymphoma were concerning. Therefore, we explored a new therapeutic approach that is simultaneously effective against both diseases. Combination therapy of lenalidomide (LEN) and rituximab (RIT) gained indication for follicular lymphoma and MALT lymphoma recently. LEN is also a key drug in MM treatment. Both diseases in our patient were effectively treated with the combination of LEN, RIT, and dexamethasone. With this combination therapy, we expect a prognostic improvement in concomitant MM and B cell lymphoma cases.

## 1. Introduction

Rarely the simultaneous onset of B cell lymphoma and multiple myeloma (MM) has been reported. One study reported that six of 4165 patients with B cell lymphoma developed MM, and one of 804 patients with MM developed B cell lymphoma [1]. There is no standard therapeutic regimen for such patients. Despite both malignancies being derived from mature B cell, the characteristics of B cell lymphoma and MM are very different. The specific therapy for each malignant tumor is administered to the patients with two types of B cell malignancies. However, their prognosis is usually poor. There has been a previously reported case of a patient with the simultaneous onset of smoldering MM (SMM) and mucosa-associated lymphoid tissue (MALT) lymphoma [2]. SMM does not typically require treatment, as with monoclonal gammopathy of undetermined significance (MGUS). However, the patient's SMM deteriorated after complete remission of the MALT lymphoma was achieved by disease-specific treatment. In this case, the patient had the simultaneous onset of MALT lymphoma and MGUS. MGUS is also considered a preneoplastic condition of malignant lymphoma (ML). In fact, 1.2% of the patients with MGUS developed ML in a long-term follow-up study [3]. Environmental and genetic factors may modify a clonal, benign proliferation into a ML. We suspect that MGUS itself also has a possibility to worsen progress of ML in the merger case of MGUS and ML, even if only a little.

The combination therapy of CD19- and B cell maturation antigen (BCMA)-chimeric antigen receptor (CAR) T cells was effective for the treatment of the patients with concomitant onset of B cell lymphoma and MM [4]. However, the CAR T therapy is costly and is not covered by insurance for MM yet in Japan. For resistant and refractory low-grade B cell lymphoma, the effectiveness of the combination of rituximab (RIT) and lenalidomide (LEN) has been recently shown [5]. It is well known that the combination of LEN and dexamethasone (DEX) is a standard regimen for MM [6]. In this case, RIT, LEN, and DEX were effective for both MALT lymphoma and MGUS.

## 2. Case Presentation

**Figure 1:**
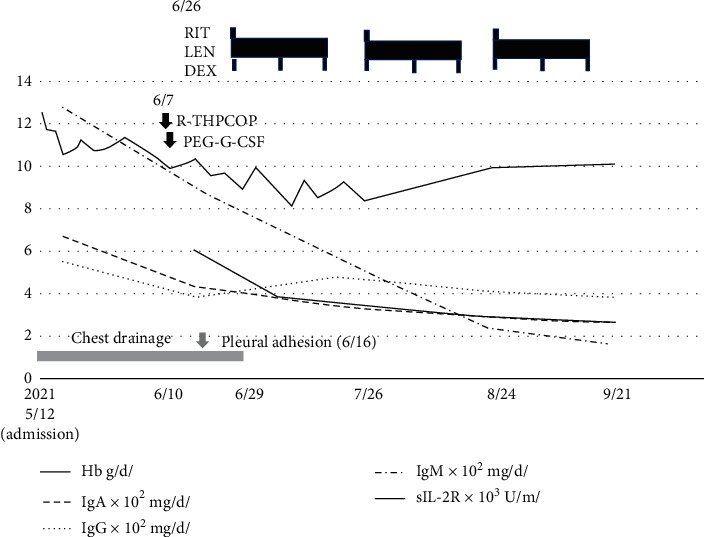
Clinical course. LEN, lenalidomide; RIT, rituximab; DEX, dexamethasone; sIL-2R, soluble interleukin-2 receptor; Hb, hemoglobin.

**Figure 2:**
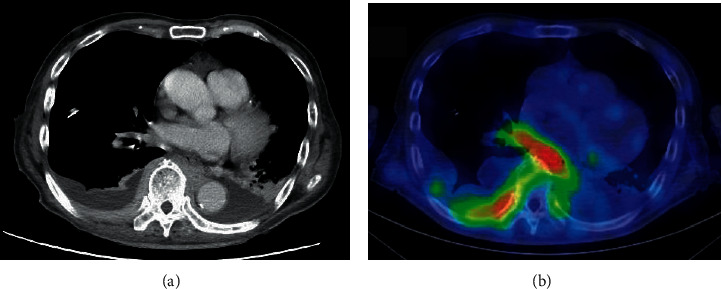
Image of pleural lymphoma. (a) Enhanced computed tomography. (b) Gallium scintigraphy.

**Figure 3:**
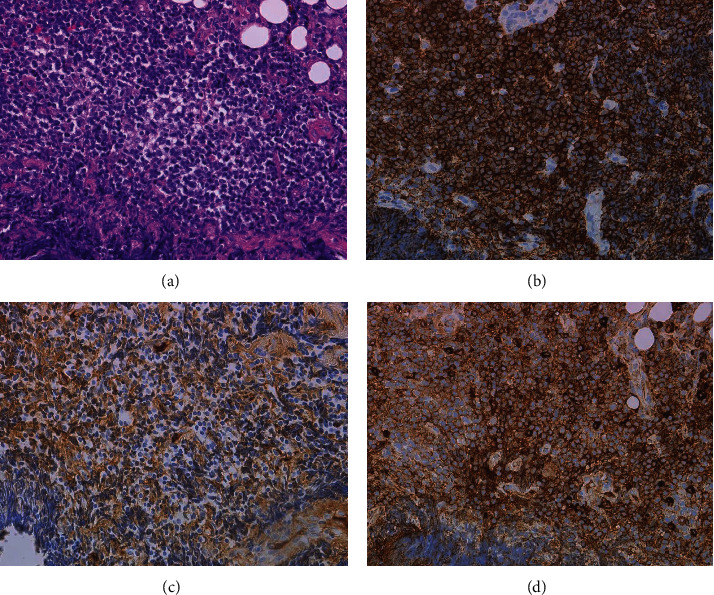
Pleural immunohistochemical staining. (a) Hematoxylin-eosin staining (×40). (b) CD20 (×40). (c) *κ* chain (×40). (d) *λ* chain (×40).

**Figure 4:**
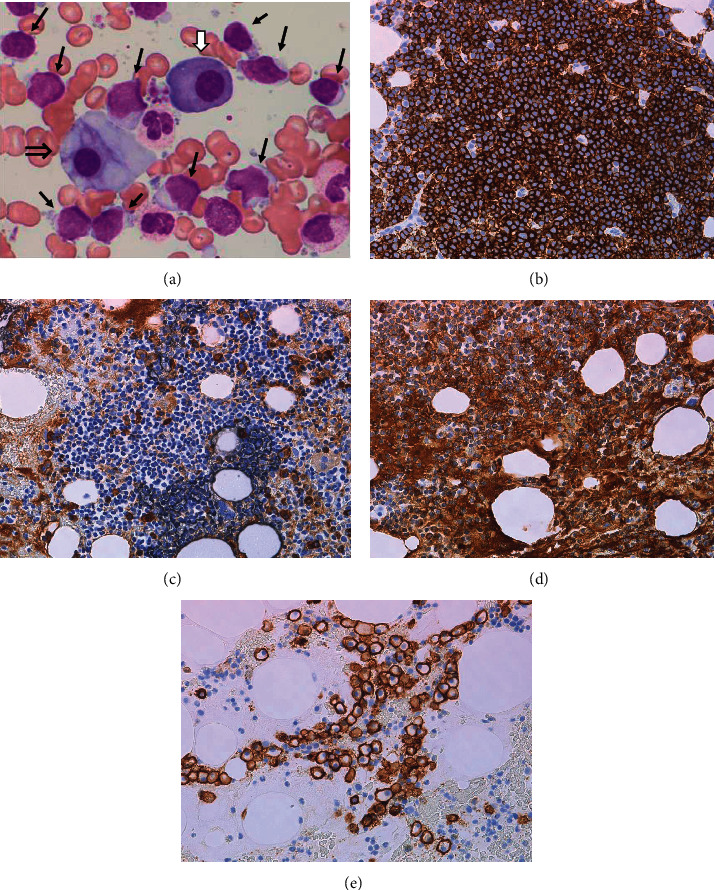
Immunohistochemical staining of bone marrow before R-THPCOP. (a) May Grunwald–Giemsa staining (×1000). (b) CD20 (×40). (c) IgA chain (×40). (d) IgM chain (×40). (e) CD138 (×40). Black arrow: lymphoma cell; white arrow: normal-sized plasma cell; double arrow: large-sized plasma cell.

**Figure 5:**
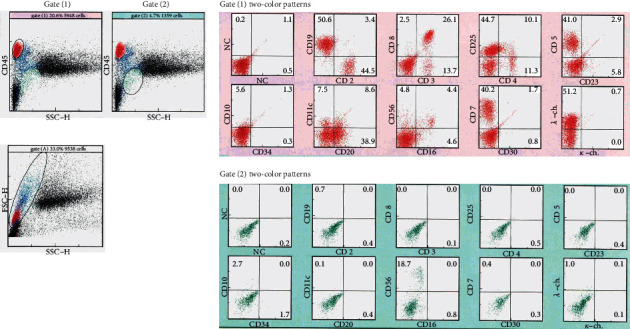
Flow cytometry analysis (CD45/SSC-H gating) of bone marrow before R-THPCOP. Two-color patterns in gate (1), and gate (2).

An 82-year-old male was hospitalized for fever and dyspnea (Figure 1). Chest radiography showed right pleural effusion. Chest drainage was carried out for the improvement of dyspnea. The pleural effusion cytopathology by Papanicolaou stain showed no malignancy. However, chromosomal karyotype analysis of the effusion demonstrated chromosome karyotype: −1, +der(?)t(?; 1)(?; p22)[5/20], XY[15/20]. Moreover, two-color flow cytometry (FCM) showed the expression of CD19, CD20, CD25, and *λ* chain, although their expression level was not high. CD25 expression is generally quite rare in MALT lymphoma. The lymphoma cells in bone marrow almost disappeared after R-THPCOP, and therapy. CD25+ cells almost disappeared after R-THPCOP; on the other hand, CD5+ cells did not decrease at all. Therefore, we thought that CD5+ cells might not be derived from lymphoma cells. We did not examine CD43 expression. FISH analyses showed no signal for IgH-CCND1 and BCL6. Computed tomography (CT) detected a pleural thickening (Figure 2(a)) in which gallium (Ga) scintigraphy showed an accumulation (Figure 2(b)). The serum soluble interleukin-2 receptor (sIL-2R) rises with the increase of the neoplastic cell in non-Hodgkin's lymphoma, reflecting the quantity of total tumor. The sIL-2R was high in our case (Table 1). We did not check the sIL-2R level just before R-THPCOP therapy. We deleted the sIL-2R level on May 21, 2021 to give an impression as if it rose after the R-THPCOP therapy (Figure 1). Primary pleural lymphoma was diagnosed because Ga scintigraphy and CT did not demonstrate any tumor lesions in other locations. Immunohistochemical staining of the pleural biopsy specimen demonstrated infiltration of *λ*-positive, monoclonal *B* cells presenting remarkable dysplasia (Figure 3(a)–3(d)). Based on morphological diagnosis and immunohistochemical staining, MALT lymphoma was strongly suspected. The expression of MIB-1 was around 30–40%. That is a little higher than that of usual MALT lymphoma. On admission, the bone marrow (BM) demonstrated that the lymphoma cells occupied about half of all nucleated cells (Figure 4(a), 4(b)). Two-color FCM of the BM expressed *λ* + CD19 + CD20 + CD25 + CD10-monoclonal malignant *B* cells, as did the pleural effusion (Figure 5). Chromosome analysis of the BM demonstrated a complex karyotype: −*Y*, del(20)(*q*11.2; *q*13.3), −1, +der(?)*t*(?; 1)(?; *p*22). Serum protein fractionation showed two *M*-peaks on admission. Immunoelectrophoresis detected the two clonal immunoglobulins of Ig*A*-*κ* and Ig*M*-*λ*. The BM smear specimen showed that the normal-sized, malignant plasma cells occupied 3%, and the large-sized, malignant plasma cell, like a foam cell, occupied 3% (Figure 4(a)). The immunostaining of the BM clot section showed that the large-sized plasma cells were IgA-positive (Figure 4(c)). However, it is not clear whether the normal-sized plasma cells express IgM because of high background level (Figure 4(d)). Both types of plasma cells were CD138-positive (Figure 4(e)). The immunostaining of the BM clot section could not clearly show the difference in the expression of the *κ* light chain and *λ* light chain because of a strong, nonspecific binding.

**Figure 6:**
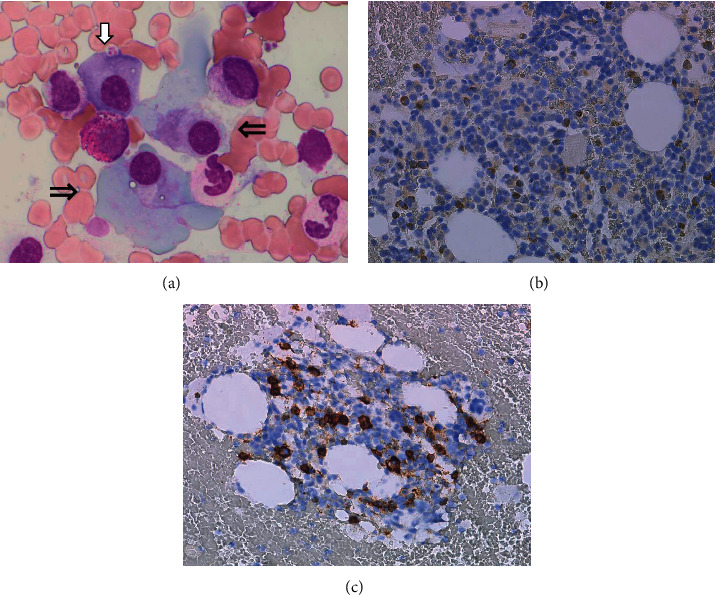
Immunohistochemical staining of bone marrow after R-THPCOP. (a) May Grunwald–Giemsa staining (×1000). (b) CD20 (×40). (c) CD138 (×40). White arrow: normal-sized plasma cell; double arrow: large-sized plasma cell.

Our case was in an advanced stage because the pleural effusion contained lymphoma cells, and he complained of dyspnea. We thought that aggressive chemotherapy for MALT lymphoma would not cause a remarkable progression of gammopathy because serum IgA and IgM levels before the treatment were not so high, and the marrow plasma cells that mainly produce these immunoglobulins were few. We treated aggressively with R (RIT)-THPCOP (pirarubicin, cyclophosphamide, vincristine, and prednisolone). After R-THPCOP therapy, the thickened pleura became slightly thinner. However, the daily pleural fluid accumulation did not diminish. Therefore, pleurodesis was performed, and the pleural drain could be removed because the output gradually decreased. The BM smear showed that the lymphoma cells had mostly disappeared (Figure 6(a), 6(b)). FCM of the BM showed the disappearance of CD20, CD25, and the remarkable decrease in CD19 and *λ* light chain expression. Chromosome analysis of the BM before R-THPCOP therapy demonstrated a complex karyotype: 45, X, −Y [10/20]/46, XY, del(20)(q11.2q13.3) [2/20]/46, XY, −1, +der(?)t(?;1)(?;p22) [2/20]/46, XY [6/20], after R-THPCOP: 45, X, −Y [8/20]/46, XY, del(20) (q11.2q13.3) [1/20]/46, XY [11/20]. The chromosome aberration detected for pleural effusion before R-THPCOP: −1, +der(?)t(?;1)(?;p22) was lost from the BM after R-THPCOP. This indicates that the lymphoma cell has the chromosome karyotype: −1, +der(?)t(?;1) (?;p22), and the two MGUS clones have the chromosome karyotypes: −Y, del(20)(q11.2q13.3). However, we were not able to identify which chromosome karyotype each MGUS clone has.

**Figure 7:**
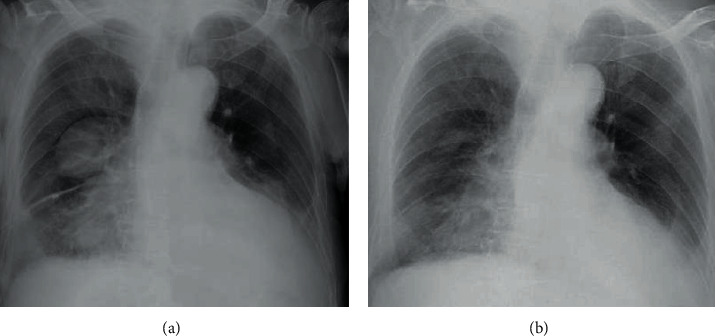
Chest radiograph. (a) Chest drainage removal date (June 28, 2021). (b) End date of 3 courses of triple therapy (September 9, 2021).

Each drug of THPCOP had been used for MM treatment in the past. After R-THPCOP, the CD138-positive cells were slightly decreased (Figure 6(c)), and the serum levels of IgM and IgA as well as IgG decreased. In comparison with the large-sized plasma cells, the normal-sized plasma cells seemed to decrease in predominance. The chest tube was able to be removed after the pleurodesis. However, the right interlobar pleural effusion recurred (Figure 7(a)). Continuous treatment for MALT lymphoma was felt to be necessary. The combination therapy of LEN, RIT, and DEX was therefore initiated. This combination became indicated for MALT lymphoma recently. It is well known that LEN is also a key drug for MM. By adding DEX to this combination therapy, we expected an improved prognosis for our patient. Specifically, LEN 10 mg/day orally for 21 days, RIT 375 mg/m^2^ intravenously monthly, and DEX 20 mg/day orally weekly were given on a cycle of 28 days. After the third cycle of triple-drug therapy, we detected a significant decline in IgM concentration, a slow decline in the Ig*A* and sIL-2R concentrations, and a slight residual of pleural effusion (Figure 7(b)).

## 3. Discussion

MALT lymphoma is thought to arise from postgerminal center memory *B* cells, which are able to differentiate into marginal zone *B* cells and plasma cells in germinal center [7]. MALT lymphomas most commonly express IgM. Differentiation to IgM-secreting plasma cells occurs in ca. 30% of MALT lymphoma [8]. It is likely that MALT lymphomas are malignancies of an IgM-only pathway of B cell development. Our case who merged MALT lymphoma and MGUS had two kinds of clonal globulins, IgM-*λ* and IgA-*κ*. We immunohistochemically detected the expression of IgM, and *λ* in MALT lymphoma. This suspected that IgM-*λ* type is derived from MALT lymphoma. The BM smear specimen showed that the normal-sized plasma cells and the large-sized plasma cell look like a foam cell. We showed that the large-sized plasma cells expressed IgA-*κ* type.

However, it is not clear whether the normal-sized plasma cells express IgM because of high background. We cannot deny that the normal-sized plasma cells might be inflammatory reactive cells. Li et al. reported the case who merged ML and three MM clones [4]. The germinal center is where somatic hypermutation of immunoglobulin and class-switch recombination occurs. MM was the neoplastic disease constructed from the cell already having received antigen stimulation in the germinal center [9]. We suppose that various genetic mutation, deletion, and amplification in MALT lymphoma might induce a mistargeting of somatic cell hypermutation and an abnormal class-switch, finally causing the multi-monoclonality of MGUS. However, it is unclear because we did not conduct a molecular analysis of our patient.

Generally, the merger cases of ML and MM were individually treated with a chemotherapy specific for each malignancy because of the lack of standard treatment guidelines. Li et al. reviewed 6 cases of coexistence of MM and ML, and 2 borderline cases with pathological and histological features of both tumors from the PubMed database [4]. Among the 6 cases, 2 patients could have clinical improvement; however, they were not followed after that. Another 2 patient relapsed one or more times. One had no further report after the third complete remission. The other patient died of infection and disease development. The 2 borderline cases died because of disease development without effect of chemotherapy. These suggest that the conventional therapeutic approach might not be able to improve the prognosis of a merger case of MM and the ML. Our case merged MGUS with MALT lymphoma not MM. MGUS does not usually require aggressive treatment. The major indication of treatment is still against symptomatic MM and MM with clinical significance but not for MGUS. Such as MGUS and monoclonal B-cell lymphocytosis, in situ lymphomas have recently been described with minimal infiltrates of clonal B cells in morphologically reactive lymphoid tissues [10]. MGUS is known to predispose to B cell malignancies [3]. Environmental factors and genetic factors have a possibility to modify a clonal benign proliferation into a malignant lymphoma [11]. *H. pylori* could be also a crucial factor to develop gastric MALT lymphoma in patients with MGUS [12]. These things suggest that MGUS itself may worsen progress of ML in the merger case of MGUS and ML, even if only a little. Therefore, some treatment is likely necessary for not only ML but also MGUS to improve their prognosis. We searched for novel regimen to simultaneously treat these two diseases with acceptable toxicity and side effects. The recent study describes future directions for improving individual management of MGUS as well as the potential for developing early treatment strategies designed to delay and prevent the development of MM [13]. We have a report that B-cell lymphoma and MM led to remission by combination therapy of CD19- and BCMA-CAR T cell at the same time. However, the CAR T cell therapy carries a very high price, and it is not covered for elderly patients in our country. Thalidomide (THD) has an effect on standard therapy-failure MALT lymphoma through the significant downregulation of nuclear NF-*κ*B expression [14]. LEN is a derivative of THD. It was revealed that single LEN administration also affected advanced MALT lymphoma [15]. The combination therapy of LEN and RIT recently gained indications for recurrent, refractory follicular lymphoma and MALT lymphoma. Furthermore, LEN is a key drug for MM treatment, and the combination of LEN and DEX is known as a standard MM regimen. Actually, in our case, the triple therapy with LEN, RIT, and DEX persistently reduced the MALT lymphoma and the levels of IgA and IgM. It is necessary to evaluate more cases to clarify whether this triple therapy can improve the prognosis in merger cases of lymphoma and MM.

## 4. Conclusion

The patients with concomitant onset of plasma cell neoplasm and B cell lymphoma have been infrequently reported. The chemotherapy specific for each disease has typically been provided sequentially. The prognosis of these patients is usually poor. We thought that therapeutic approaches which could simultaneously treat both malignancies might be important. This is the first report where the combination of LEN, RIT, and DEX could effectively treat MALT lymphoma and MGUS simultaneously. We expect this combination therapy to improve the prognosis of the cases with concomitant onset of MM and B cell lymphoma.

## Figures and Tables

**Table 1:** Laboratory data on admission.

Complete Blood Count		Biochemistry test		Serological test	

WBC	5,900/*μl*	TP	7.8 g/d*l*	C3	79 mg/d*l*
Banded neutrophil	1.0%	Alb	3.5 g/d*l*	C4	23 mg/d*l*
Segmented neutrophil	84.0%	AST	14 U/*l*	ANA	40 fold
Lymphocyte	4.0%	ALT	5 U/*l*	RF	6 IU/m*l*
Monocyte	3.0%	ALP	270 IU/m*l*	BNP	73 pg/m*l*
Eosinophil	8.0%	*γ*-GTP	16 IU/*l*	HCV Ab	(−)
RBC	426 x 10^4^/*μl*	CHE	186 U/L	HBs Ag	(−)
Hb	12.5 g/d*l*	LDH	208 U/*l*	IgG	546 mg/d*l*
Ht	37.5%	T-bil	0.6 mg/d*l*	IgA	662 mg/d*l*
MCV	88.0 f*l*	BUN	22.5 mg/d*l*	IgM	1272 mg/d*l*
Plt	24.6 × 10^4^/*μl*	Cr	1.66 mg/d*l*	sIL-2R	3190 U/ml
		UA	3.9 mg/dl		
Urinalysis		T-CHO	166 mg/d*l*	Coagulation	
Color	Yellow	TG	80 mg/d*l*	PT-INR	0.96
Protein	1(+)	Glu	109 mg/d*l*	APTT	33.4 sec
Glucose	(−)	HbA1c	5.3%	Fibrinogen	294.6 mg/d*l*
Occult blood	(+/−)	Na	137 mEq/*l*	FDP	6.5 *μ*g/m*l*
		K	3.5 mEq/*l*	D-dimer	3.0 *μ*g/m*l*
		Cl	102 mEq/*l*		
		CRP	0.17 mg/d*l*		

## Data Availability

All the data are available in the hospital's medical records.
